# Assessment of the inclusion of a feed additive of sodium humate derived from freshwater sapropel in diets for broiler chickens

**DOI:** 10.14202/vetworld.2023.2029-2041

**Published:** 2023-10-07

**Authors:** Liga Proskina, Dace Barzdina, Anda Valdovska, Irina Pilvere, Ilze Vircava, Sallija Cerina, Sandijs Meskis

**Affiliations:** 1 Institute of Economics and Finance, Faculty of Economics and Social Development, Latvia University of Life Sciences and Technologies, Jelgava, Latvia; 2 Institute of Animal Science, Faculty of Agriculture and Food Technology, Latvia University of Life Sciences and Technologies, Jelgava, Latvia; 3 Institute of Food and Environmental Hygiene, Faculty of Veterinary Medicine, Latvia University of Life Sciences and Technologies, Jelgava, Latvia; 4 Research Laboratory of Biotechnology, Latvia University of Life Sciences and Technologies, Jelgava, Latvia; 5 Institute of Soil and Plant Sciences, Faculty of Agriculture and Food Technology, Latvia University of Life Sciences and Technologies, Jelgava, Latvia; 6 Department of Plant Breeding and Agroecology, Institute of Agricultural Resources and Economics, Latvia

**Keywords:** broilers, carcas yield, growth performance, ileum microbiota, sapropel, sodium humate

## Abstract

**Background and Aim:**

Poultry production is the fastest growing livestock industry in the world, as the rapid growth of and efficient absorption of feed by poultry ensure the production of poultry meat with a relatively low carbon footprint. Seeking new ways to increase livestock productivity as well as poultry product quality, the number of research studies on the use of humic substances of various origins in livestock farming has increased significantly, emphasizing the role of feed additives derived from local resources. The unique capability of humic substances to improve metabolic processes allows the immune protection of the bird body to be strengthened and production efficiency to be increased. This study aimed to identify the effects of sodium humate (NaHum) on the growth performance of broiler chickens and selected blood and ileum microbiota parameters.

**Materials and Methods:**

Dietary research was conducted 2 times under production conditions in a poultry facility of a commercial company, with 210 1-day-old, unsorted broiler chickens of both sexes (Ross 308). The broiler chickens were fed with standard commercial feed, the rearing period of 35 days, and slaughtered on day 36. Sodium humate additive was added to drinking water for the research groups of broilers in period from 8^th^ to 35^th^ day of life, 25 mL (Group 1, n = 2 × 35) and 50 mL (Group 2, n = 2 × 35) per liter of drinking water. Sodium humate contained an average of 4.48% dry matter, a kilogram of dry matter containing 104.3 g of crude protein, 3.6 g of crude fiber and 0.9 g of crude fat, 14.3 MJ of metabolic energy, and 5.8 MJ of energy for live weight gain, as well as a very high content of crude ash −759.8 g, including 4.2 g Ca, 4.2 g Na, and 4.81 g Fe, the dry matter digestibility of NaHum was 87.0%, and the absorption capacity of dry matter was 113.2%., the pH level was 13.0, i.e., alkaline. At the end of the dietary research, the productivity and economic efficiency of the research groups of broilers were calculated by live weight gain, carcass weight, feed conversation ratio, and blood and intestinal samples of broilers were analyzed to identify the effects of NaHum on the growth performance and health status of broilers.

**Results:**

Dietary research found that adding 25 mL/L and 50 mL/L of NaHum to drinking water for the broiler chickens increased their live weights at the selling age, average live weight gains by 3.06–3.93%, and carcass weights by 5.07–6.06%, while feed conversion increased in terms of both live weight (1.5 and 1.51) and carcass weight (1.84 and 1.86) compared with the control group. The best economic performance in terms of the economic efficiency index and the cost index (CI) was found in Group 1, which was fed with the NaHum additive at an intake rate of 25 mL/L. The NaHum additive modulated the ileal microbiota and metabolic processes in the broiler body. At the same time, a significant decrease in the levels of total protein, alkaline phosphatase and phosphorus (P) in blood was found in the research groups.

**Conclusion:**

Considering the positive effects of NaHum derived from freshwater sapropel on the productivity and economic efficiency of broiler chickens, the NaHum feed additive should be further investigated on a larger scale to obtain results that could reasonably be used in practice. This study concluded that a decrease in P levels in the blood was observed when NaHum was added to the drinking water; therefore, it is important to continue the research to draw reasonable conclusions on the effects of NaHum in liquid form on the health performance of farm animals.

## Introduction

Safe, nutritious, and accessible food is the cornerstone of food security [1], and the growing demand for food is an important challenge to be faced in the coming decades [2–4]. It is forecasted that global food production needs to increase by at least 70% by 2050 to feed the growing global population; therefore, industrial-scale agricultural activities such as poultry production could be a potential priority [5]. The rapid growth and efficient absorption of feed by poultry ensures the production of poultry meat with a relatively low carbon footprint [6–8]. A significant decrease in the demand for rice, an increasing share of palm oil in the global fat and oil markets, and a continued shift to poultry as the dominant form of meat consumption are predicted by 2050 [9]. Globally, the poultry industry has developed dynamically and experienced significant achievements in recent decades [10], as poultry meat has become the most consumed livestock product in the world [11]. Global meat consumption per individual is expected to increase significantly: Up to 9 kg in 2030 and up to 18 kg in 2050, of which 12.5 kg or 69% will be poultry meat [12, 13]. The European Union (EU) is one of the world’s largest producers of poultry meat and a net exporter of poultry meat products, producing approximately 13.4 million tons of poultry meat annually [14]. Conventional poultry production systems, which use fast-growing crossbreeds or broilers, face three critical constraints: (1) The genetics of one-day-old chickens, or their potential and quality; (2) the feeding process or feed quality; and (3) chicken health and care [15]. Intensive poultry farming makes the problem with poultry diets urgent. Therefore, research studies on cheaper and accessible feed and biologically active principles that can increase poultry productivity and reduce the cost of production are important [16, 17].

Since 2000, there has been a growing interest in the use of humus and its biologically active principles in livestock farming. Several scientific studies on feed absorption efficiency in poultry farming [2, 6, 8, 10, 17–22] have shown a positive effect on the quality of meat and eggs and livestock health. Some research studies have found that various feed additives and biologically active principles included in the poultry diet increased the productivity of poultry, increased feed absorption efficiency, and consequently reduced production cost per kilogram of meat produced [10, 23]. Many researchers have found improvement in livestock growth and feed conversion, as well as reduction in livestock mortality after adding humic substances to feed [24–26]. Freshwater sapropel, which is formed from the remains of aquatic vegetation, contains living organisms, plankton, and soil humus particles; as it is made up of a significant amount of organic and mineral matter, it could be considered an important source of humic substances [27]. In some cases, sapropel contains up to 50%–60% organic matter and up to 30%–50% mineral matter [28]. Furthermore, economic growth requires wider use of local resources; therefore, effective use of materials available in nature is a worldwide priority [29]. One use of sapropel is to derive extracts from it, mostly from the humic substances contained in it, as their use in diets for farm animal’s increases weight gain, improves blood hematological parameters, and strengthens the immune system of the animals [30]. In addition, humic substance preparations make agricultural production ecologically clean, which favorably stands out against the background of increasing environmental pollution caused by the production of various chemical industry preparations [18, 31, 32]. Several researchers have identified the most important properties of humates [33]: (1) Antioxidant properties that have a strong ability to maintain a chemical balance in the body [34]; (2) antiviral activity that blocks the entry of a virus into the cell and interferes with its replication [35, 36]; and (3) detoxifying and hepatoprotective effects – humic acids bind and remove heavy metals from the body. Long-term use of humic substances has a beneficial effect on liver function [35]; (4) antibacterial effects because humic substances have an antibacterial effect on various pathogenic microorganisms. Humic acids neutralize pathogenic microflora in the intestinal tract and suppress pathogenic microorganisms, stimulate the growth of natural intestinal microflora, improve protein digestion and absorption of calcium (Ca), trace elements, and nutrients [36]; (5) enterosorption [37]. Humic acids form a thin gel-like film on the mucous membrane of the gastrointestinal tract, which protects the body from infections and toxins. During the use of humic acids by the body, only toxins and surplus minerals are removed from the body, yet useful trace elements needed by the body are not lost. Scientific and economic experiments with broiler chickens found that the poultry fed with extruded sapropel pellets achieved a higher weight gain [38]. During dietary research with laying hens in Latvia, the effect of sapropel as a feed additive was examined over a period of 150 days, and it was found that eggshell thickness increased [39].

Therefore, to further develop and increase the efficiency of poultry farming, innovative solutions are needed to achieve more efficient use of feed by using biologically active principles of natural origin that are derived from local resources and raw materials. The results of this study will contribute to and supplement the knowledge and findings available in research studies on the application of freshwater sapropel and the products derived therefrom in practice.

The research put forward a hypothesis – the inclusion of a feed additive of NaHum derived from freshwater sapropel in diets for broiler chickens increases the productivity of broiler chickens and modulates the ileum microbiota and metabolic processes in the broiler body. This study aimed to identify the effects of sodium humate (NaHum) on the growth performance of broiler chickens and selected blood and ileum microbiota parameters.

## Materials and Methods

### Ethical approval

This study complied with the requirements of the Law of the Republic of Latvia (2007) “On the European Convention for the Protection of Animals Kept for Farming Purposes and the Protocol” [40]. The dietary research did not involve any activities that would require an ethical statement. The dietary research was conducted in cooperation with an agricultural company, Valmiera Agro Ltd., under production conditions at its registered broiler facility in Latvia, Vidzeme region, where the NaHum additive derived from sapropel was included in diets for broilers. Observations and data collection were performed as part of routine work at the facility. Blood, gut, and muscle samples from the broilers were collected at the end of the research when the broilers were slaughtered for commercial purposes at a certified slaughterhouse. According to EU Directive 2010/63/EU Article 1, Paragraph 5, Point a), an approval from the ethics commission is not required for non-experimental agricultural practices [41].

### Study period and location

The dietary research was conducted from May to June in 2021 and from May to June 2022, in cooperation with an agricultural company Valmiera Agro Ltd. (Vidzeme region, Latvia), under production conditions at its registered broiler facility. The duration of broiler rearing was 35 days, the broilers were slaughtered and processed on the 36^th^ day according to the standard production period on the farm.

### Freshwater sapropel reserves and the extraction site

Freshwater sapropel is a gel-like substance containing colloidal organic matter and is essentially composed of zooplankton and phytoplankton, with diatom skeletons (diatoms), green algae, cyanobacteria, foraminifera, radiolaria, dinophyte algae, bacteria and the remains of aquatic plants or organisms being most often found. In addition to organic material, sapropel may also contains mineral particles such as sand, clay, Ca carbonate, and other compounds [42]. In the territory of Latvia, sapropel is found in most of the lakes and in more than a third of bogs where the layer of it can reach a thickness of up to 20 m. In 2021, sapropel reserves were estimated at 732.4 million m^3^ (according to Latvian Environment, Geology and Meteorology Centre data) [43], yet the total deposits could exceed 2 billion m^3^ [44]. Although in the first half of the last century sapropel was mainly used for soil fertilization as a soil conditioner or additional fertilizer [45], today it is being examined by materials science and is used in cosmetics, medicine, and many other industries.

This research used organosilicate sapropel extracted from Lake Bizas located in Latvia, Kraslava municipality, Andrupene parish. Lacustrine sediments overlie glacial and glaciolacustrine sediments. As the climatic conditions changed in the Holocene, the amount of organic matter in the lake increased. The surface area of the lake is 142.29 ha, and the sapropel layer is 0.4–2.7 m thick; the lake could be classified as a through-flow lake. According to the main type, the lake could be classified as eutrophic (rich in nutrients). The sapropel deposits of Lake Bizas are estimated at 6.5 million m^3^, of which 4567.8 thousand m^3^ or 1 190.72 thou.t (at moisture content W = 60%) represent the reserves to be extracted [46].

### Production and composition of freshwater sapropel and a NaHum additive

NaHum was extracted from freshwater organosilicate sapropel, where the amount of organic matter in an absolutely dry sample was 49%. The abs. moisture content of sapropel at the time of extraction reached 95%. Sodium humate was produced according to a methodology [47] developed by the Humus Substances Association. A solution of 100 g abs. dry sapropel in 2L of 0.5M NaOH solution was heated to 85°C on a hotplate stirrer and continuously stirred at a speed of 850 rpm (revolutions per minute) 4 h. The solution containing humates was separated from the water-insoluble part after centrifugation at 2713 × *g* for 15 min. The samples were stored in closed containers at 4°C, in the dark, until being fed to broilers. The chemical composition of NaHum included in diets for broilers is presented in Table-1. The NaHum obtained was alkaline at pH 13, contained macro and micro elements Ca, potassium (K), phosphorus (P), and sodium (Na), magnesium (Mg), Iron (Fe), etc., and the iron content was high at 4.81%. The crude protein content was high at 104.3% and dry matter digestibility was 82.3%.

**Table-1:** Chemical composition of complete feed and NaHum fed to broiler chickens.

Composition of feed	Starter feed 1^st^–10^th^day	Grover feed 11^th^–28^th^day	Finisher Feed 29^th^–35^th^day	NaHum
Dry matter, %	91.05	89.33	88.32	4.48
In dry matter				
Crude protein, %	24.90	23.03	21.18	10.43
Crude fiber, %	2.89	3.75	3.93	0.36
NDF, %	8.90	11.75	11.61	-
ADF, %	4.20	6.06	5.84	-
NEL, MJ/kg	8.28	8.13	8.14	-
NEM, MJ/kg	9.20	9.03	9.05	-
NEG, MJ/kg	6.18	6.01	6.03	0.58
TDN/DDM, %	85.63	84.18	84.35	82.3
DMI, %	13.48	10.21	10.34	113.2
RFV	894.99	666.44	675.85	-
ME for poultry, MJ/kg	15.46	15.59	15.92	1.43
OMD, %	94.88	90.44	92.07	87.0
Fat, %	4.58	7.19	8.39	0.09
Crude ash, %	6.84	6.11	5.49	75.98
Ca, %	1.05	0.89	0.73	0.42
P, %	0.77	0.75	0.64	0.08
K, %	1.11	0.99	0.91	0.26
Mg, %	0.26	0.26	0.23	0.17
Cu, mg/kg	22.08	28.63	24.05	0.01
Na, g/kg of dry matter	-	-	-	4.2
Zn, g/kg of dry matter	-	-	-	0.09
Mn, g/kg of dry matter	-	-	-	`0.06
Fe, g/kg of dry matter	-	-	-	4.81
Starch, %	45.45	44.52	43.94	-
pH	-	-	-	13.0

NDF=Neutral detergent fiber, ADF=Acid detergent fiber, NEL=Net energy of lactation, NEM=Net energy for maintenance, NEG=Net energy for gain, DDM=Digestible dry matter, DMI=Dry matter intake, RFV=Relative feed value, OMD=Organic matter digestibility, Ca=Calcium, P=Phosphorus, K=Potassium, Mg=Magnesium, Cu=Copper, Na=Sodium, Zn=Zinc, Mn=Manganese, Fe=Iron, TDN=Total digestible nutrients, ME=Metabolizable energy, MJ=Megajoule, NaHum=Sodium humate

### Experimental design, birds, and diets

For the dietary research, 2 × 105 1-day-old, unsorted broiler chicks of both sexes (Ross 308), with an average live weight of 42.55 ± 3.33 g, were purchased from the JSC Putnu fabrika Kekava, which is the leading full-cycle poultry meat production company in Latvia. Broiler chicks were randomly divided into three groups: Control (Group C, n = 2 × 35, average live weight 41.7 ± 3.46 g) and two research groups (Group 1, n = 2 × 35, 43.5 ± 3.09 g and Group 2, n = 2 × 35, 42.7 ± 3.24 g). The chickens were kept on coniferous wood-chip bedding; the poultry density, according to the provisions of Council Directive 2007/43/EC of June 28, 2007 [48], did not exceed the maximum live weight density of 33 kg/m^2^ or 12 chickens per 1 m^2^. The chickens were reared according to the technological instructions developed for Ross 308, which required controlling the lighting, temperature, animal hygiene, feeding, and watering regimes [49]. Access to water and feed was *ad libitum*. At the beginning of the dietary research, the broiler chickens of all the groups were fed only basic feed and watered with clean water for the first 7 days. From the 8^th^ to the 14^th^ day of life, NaHum was added to drinking water for the broiler chickens of the research groups (Group 1 and Group 2) at an increasing rate, thereby accustoming the chickens to NaHum. From day 15 onward, NaHum was added to drinking water at a constant rate: 25 mL/L of water for Group 1 and 50 mL/L of water for Group 2.

### Composition of NaHum and feed

All the broilers were fed the same commercially prepared complete feed mixture, based on the periods of chick development (starter period from 1 to 10 days: grower period from 11 to 28 days and a finisher period from 29 days to the end of fattening). The complete feed mixture fed to the broilers in all the periods represented a premix consisting of corn, soybean meal, wheat, triticale, vegetable oil, rapeseed cake, Ca carbonate, monocalcium phosphate, amino acid (DL-methionine, L-lysine), Na chloride, sodium sulfate, sodium bicarbonate, and wheat flour.

All feed and water samples were examined at the Research Laboratory of Biotechnology of Latvian University of Life Sciences and Technologies (LBTU) (accreditation No. LATAK-T-168) to identify the amount of dry matter and the parameters of chemical composition of the nutrients included in the feed in terms of percentage and volume. The chemical parameters were identified according to generally accepted standards or calculation methods. Sodium humate tests were performed before each replication. The samples were tested for dry matter (oven-drying, according to LVS EN ISO 6498); crude protein (Kjeldahl method, LVS EN ISO 5983-2); crude fiber content (cellulase method, ISO 5498); crude fat content (Soxhlet extraction, ISO 6492); crude ash content (ISO 5984); starch content (polarimetric method, LVS EN ISO 10520); and elements (Ca, K, Mg, Na, Zinc, Copper, Manganese, Fe) using the atomic absorption spectrometry method (LVS EN ISO 6869) and P (photometric method, ISO 6491). All chemical test results were reported on a dry matter basis.

### Assessment of productivity and economic efficiency of broiler chickens

Feed intake (FI), calculated as a difference between the amounts of feed fed and not eaten by the broilers (recorded every day), and water intake (WatI) (recorded every day) were identified to assess the effects of the NaHum additive on the productivity and economic efficiency of broilers.

To compare the digestibility and palatability of different feedstuffs, relative feed value (RFV) was calculated according to the following equation:


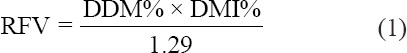


where DDM% - digestible dry matter (%); DMI% - dry matter intake (%). The broilers were weighed once a week during the research period to identify changes in broiler live weight (BW). The growth of broilers, the weights of the carcass, and parts of the carcass were identified using CAS company scales (Model SW-1, accuracy ± 0.1 g, Retail weighing solution™, East Rutherford, NJ, USA). Average daily weight gain (DWG) (g) as well as a broiler survival rate (%) were calculated once a week throughout the fattening period and at the end of the study. Feed conversion rate (FCR) is an important indicator that allows production costs to be tracked for increasing broiler productivity, and this was calculated per BW for each of the research groups at the end of the research (i):


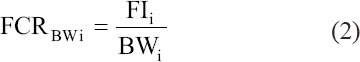


At the end of the research, after processing the broilers in the slaughterhouse (stunning, bleeding, scalding, plucking, and evisceration), carcass yield (CY) was identified for all the broilers. To identify differences in weights of parts of the carcass and tissue, 10 broilers (males) from each group with a similar live weight (±5%) were divided into carcass and tissue parts. Carcass yield (%) was calculated as a ratio of eviscerated carcass weight to live body weight at slaughter:


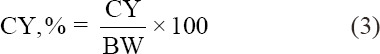


The ratio of individual parts of the carcass (chest muscles, wings, muscle tissue, bone tissue, and skin) to CY was also determined. To identify the real volume of broiler products, a FCR per CY was calculated as follows:


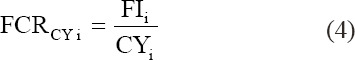


The European production efficiency factor suggested by Aviagen [49] was used to calculate broiler productivity indicators using the following equation:


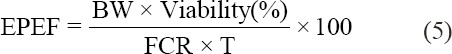


where T is the research period in days. The second important indicator is the European broiler index (EBI), which was calculated according to the following equation [50]:


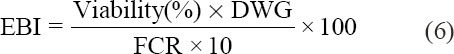


To identify economic efficiency, an approach suggested by Martins *et al*. [51] was additionally applied to calculate the ratios of feed cost (FC) to live weight and carcass weight using the following equations:


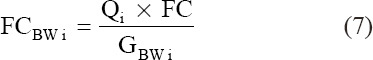



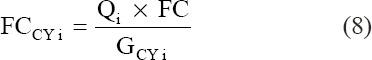


where Q_i_ is the amount of feed consumed by the research group and G_i_ is the BW or CY in each of the research groups.

Broiler diet costs and feed consumption were identified separately during the feeding phase and were calculated at the end of the study. The average market prices of poultry feed purchased by the farm in Latvia in 2021 and 2022 were considered for an analysis of FCs. An economic efficiency index (EEI) and a cost index (CI) were calculated according to Martins *et al*. [51]:


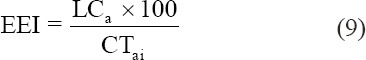



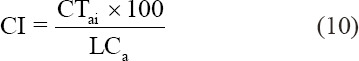


where LC_a_ is the lowest FC per kg of live or carcass weight for the research group and CT_ai_ is the FC for the respective group.

### Blood sample collection and testing

At the end of the dietary research (day 36), the broilers were not fed for 12 h before slaughter. Blood was collected from 6 randomly selected broilers from each group per treatment. The blood samples were taken from a wing vein in sterile tubes, stored in a +4°C environment and delivered to the laboratory within 4 h. At the laboratory of LBTU, serum was derived from blood samples by centrifuging it at 3023 × *g* for 20 min in a “Sigma, 3-16L” centrifuge (Osterode, Germany). The serum was stored in 1.5 mL Eppendorf tubes at −20°C until further investigation. The following characteristics were identified for the serum by employing the absorption photometry method: Glucose (GLU), total cholesterol (TC), total protein (TP), albumin (ALB), alkaline phosphatase (ALP), P, Ca, gamma-glutamyltransferase (GGT), and triglycerides using a clinical chemistry analyzer (Mindray BS-380, Shenzhen Mindray Bio-Medical Electronics Co. Ltd., Shenzhen, P. R. China). For hematological parameters, blood smears were prepared immediately after blood withdrawal. The labeled blood smears were air-dried, fixed with methanol, and stained with Giemsa in the laboratory. By counting 100 leukocyte cells in the blood smear, the leukocytes, monocytes, and lymphocytes were calculated in percentage terms.

### Fecal sample collection and testing

A part of the ileum (from the pancreo-biliary part to Meckel’s diverticulum) obtained from each intestinal tract of a broiler was tested, tied at both ends, placed in sterile bags, stored in a +4°C environment, and delivered to the laboratory within 4 h. Tests on fecal microbiota were performed within 2 h of the samples being delivered to the laboratory. The fecal contents were used to identify the counts of coliforms, *Lactobacillus* spp. and *Clostridium* spp. Initial and serial dilutions of the samples were made in peptone saline (Maximum Recovery Diluent, Biolife, Milan, Italy) according to ISO 6887-1:1999. For the isolation and enumeration of the *Enterobacteriaceae* bacteria, Violet Red Bile GLU agar, Biolife was used and tested according to ISO 21528-2:2007. The isolation and enumeration of *Lactobacillus* spp. were performed using De Man–Rogosa–Sharpe agar with Tween 80 (Biolife, Milan, Italy) in accordance with the medium manufacturer’s instructions. The incubation was realized at 36°C ± 1°C, for 72 h ± 2 h. The research confirmed the most typical colonies by performing Gram staining and catalase test. Tryptose sulfite cycloserine agar, Oxoid (Hampshire, United Kingdom) was used for the isolation of *Clostridium perfringens* bacteria. The fecal dilution plates prepared were incubated under anaerobic conditions at 36°C ± 1°C, for 24 h ± 1 h in a BD Gas-Pak EZ container system with BD BBL CO_2_ generators and indicators (Franklin Lakes, NJ, USA). The results obtained were calculated and expressed as log10 colony-forming units per gram of fecal contents.

### Statistical analysis

The results were statistically analyzed with RStudio software ver. 4.1.2. (https://www.npackd.org/p/r/4.1.2), one-way analysis of variance being applied. The significance of differences between the mean values was evaluated using the Tukey HSD test, with a significance level of 95%. Data are presented as means ± standard deviation.

## Results

### Changes in the productivity and economic efficiency of broiler chickens

Relative feed value was calculated for the feed of each broiler growth period (starter, grower, and finisher). The highest RFV value was found in starter feed – 894.99. It gradually decreased in the later stages of the fattening period to 666.44 and 675.85, respectively. During the research period, the broiler chickens of the control group consumed the most feed – 260.23 kg. The broiler chickens of Group 1 consumed the least amount of feed – 252.64 kg, while the broiler chickens of Groups 1 and 2 consumed an average of 0.105 kg of feed per day. The broiler chickens of Group 2 consumed the most water during the research – 546.83 L, and if measured as per broiler per day, the broiler chickens of this group consumed the most, or 0.227 L. During the entire dietary research, 7.218 L of NaHum was added to drinking water. According to the methodology, 25 mL/L and 50 mL/L NaHum was added to drinking water daily; during the research period Group 1 broiler chickens consumed a total of 2.400 L NaHum, and Group 2 broiler chickens twice as much – 4.818 L NaHum (Table-2).

**Table-2:** Feed, water, and NaHum consumed by the groups per broiler per day.

Broiler group	Control group (n = 70)	Group 1 (n = 70)	Group 2 (n = 70)
Total feed consumed, kg	260.21	252.68	253.30
Feed consumed per broiler per day, kg	0.106	0.105	0.105
Total water consumed, L	473.08	491.06	546.83
Water consumed per broiler per day, L	0.195	0.209	0.227
Total NaHum consumed, L	-	2.400	4.818
NaHum consumed per broiler per day, L	-	0.034	0.069

The numerically highest average live weight (Table-3) at the beginning of the research was found in Group 1 (201.47 ± 24.58 g); at the end of the research, the broiler chickens of Group 2 had the numerically highest average live weight (BW) (2499.43 ± 260.80 g). However, differences in live weight between the groups were not significant.

**Table-3:** Productivity indicators of broiler chickens.

Indicator	Control group (n = 70)	Group 1 (n = 70)	Group 2(n = 70)
Live weight at the beginning of the dietary research, day 8, g	198.24 ± 28.5	201.47 ± 24.58	200.12 ± 27.81
p-value	-	0.61	0.83
Live weight at the end of the dietary research, day 35, g	2411.5 ± 296.66	2478.4 ± 301.35	2499.4 ± 260.80
p-value	-	0.20	0.07
Live weight gain per day, g	67.7 ± 8.43	69.6 ± 8.54	70.2 ± 7.41
p-value	-	0.21	0.07
Feed conversion to live weight	1.61	1.48	1.51
Survival rate, %	94.44	97.14	95.71
Carcass weight of a slaughtered broiler, g	1921.5 ± 265.59^a^	2016.7 ± 246.81^b^	2036.3 ± 234.77^b^
p-value	-	0.03	0.01
Feed conversion to carcass weight	2.02	1.84	1.86
Carcass yield, %	77.46 ± 3.57^a^	79.22 ± 3.60^b^	79.24 ± 4.47^b^
p-value	-	0.005	0.012

^a,b^Different letters for the same indicator indicate significant differences between the groups, (p ≤ 0.05), data are presented as means ± SD. SD=Standard deviation

An analysis of FCR_BW_ (equation 2) revealed that the lowest feed consumption per kg of live weight was found in research Group 1 (1.48). The results were similar in research Group 2 (1.51), the FCR_BW_ being higher in the control group (1.61); however, no significant differences were found. The highest viability of broiler chickens (97.1%) was observed in Group 1. Although the development indicators were higher for the broiler chickens of Group 1, a numerically higher (p > 0.05) average DWG was observed in Group 2 at 70.2 ± 7.41 g.

However, an analysis of broiler chicken carcass weight and yield (Table-3) revealed significant differences between the control and research groups (p ≤ 0.05). The highest carcass weight (2036.3 ± 234.77 g), the highest CY (79.24 ± 4.47%) was found in Group 2, which was watered with 50 mL/L NaHum. Accordingly, the differences in FCR_CY_ or feed consumption per kg of carcass weight between the groups were much more significant. An analysis of FCR_CY_ (equation 3) revealed the best performance in Group 1, with 1.84 kg of feed consumed to produce a kg of carcass weight. Similar performance was also found in Group 2, as 1.86 kg of feed was needed to produce a kg of carcass weight. In contrast, FCR_CY_ was 2.02 kg in the control group, which was 0.16–0.18 kg more than that in the research groups. The calculated indicators proved that adding NaHum to drinking water for broiler chickens reduced feed consumption per unit of production, increasing live weight and CY.

An analysis of the weights of parts of the carcass and internal organs (Table-4) revealed no significant differences between the groups; however, a positive trend was observed in the research groups compared with the control group. The highest proportion of breast musculature was found in the broiler chickens of Group 2 (30.37 ± 1.78%); similarly, the proportion of bone tissue was the lowest (21.17 ± 1.12%) in the broiler chickens of this group, which indicated the higher productivity of broiler chickens whose drinking water was supplemented with NaHum.

**Table-4:** Distribution of carcass parts and internal organs of broiler chickens, %.

Carcass parts and internal organs	Control group (n = 12)	Group 1 (n = 12)	Group 2 (n = 12)
Chest musculature (tenderloin)	29.37 ± 3.34	29.61 ± 2.33	30.37 ± 1.78
Other musculature*	36.48 ± 3.53	37.52 ± 3.38	36.92 ± 1.79
Bones	21.83 ± 1.81	21.65 ± 1.89	21.17 ± 1.12
Skin	12 ± 1.03	11.22 ± 1.23	11.55 ± 0.75
Liver	2.57 ± 0.24	2.46 ± 0.24	2.51 ± 0.27
Heart	0.75 ± 0.07	0.71 ± 0.07	0.74 ± 0.04

* Legs and wings musculature

The highest proportion of liver (2.57 ± 0.24%) and heart (0.75 ± 0.07%) in the total weight was found in the control group. This might indicate a more intensive metabolism during feed processing. In contrast, the proportion of heart and liver was slightly lower in Groups 1 and 2; however, no significant differences were found, and it could be assumed that the NaHum additive did not cause problems in metabolic processes.

The best economic performance in terms of the EEI (EEI_BW_, EEI_CY_) and the CI (CI_BW_, CI_CY_) and in terms of both live weight and carcass weight was found in research Group 1, which was watered with 0.25 mL/L NaHum (Table-5).

**Table-5:** Indicators of economic efficiency for the broiler groups.

Indicator	Control group (n = 70)	Group 1 (n = 70)	Group 2 (n = 70)
EPEF	407.64	458.80	451.86
EBI	400.57	450.75	444.14
FC per research broiler	2.15	2.22	2.37
FC_BW_	0.90	0.89	0.95
FC_CY_	1.18	1.10	1.17
EEI_BW_	99.81	100.00	94.14
EEI_CY_	93.38	100.00	94.26
CI_BW_	100.19	100.00	106.22
CI_CY_	107.08	100.00	106.09

EPEF=European production efficiency factor, EBI=European broiler index, CI_CY_=Cost index of carcass yield, CI_BW_=Cost index of live weight, EEI_CY_=Economic efficiency index of carcass yield, EEI_BW_=Economic efficiency index of live weight, FC_CY_=Ratio of feed cost to broiler carcass weight, FC_BW_=Ratio of feed cost to broiler live weight, FC=Feed cost

### Hematology, blood serum chemistry, and ileum microbiota composition

The hematological parameters of broiler blood are presented in Table-6 [52, 53]. At the end of the dietary research, the blood inflammatory cell count results were mainly within the reference limit. A higher count of heterophils (%) was found in the control group, while a higher count of eosinophils (%) – in Group 2.

**Table-6:** Hematological and biochemical parameters for broilers fed with the NaHum additive at the end of the research.

Parameters	Control group (n = 12)	Group 1 (n = 12)	Group 2 (n = 12)	Reference	p-value Control/Group 1	p-value Control/Group 2
Hematological parameters Jain [52]					x	x
Heterophils (%)	47.67 ± 4.49	38.33 ± 2.17	41.67 ± 3.34	15–40	0.172	0.458
Eosinophils (%)	4.00 ± 0.89	6.17 ± 1.14	7.17 ± 0.70	1.5–6	0.257	0.071
Monocytes (%)	2.50 ± 0.62	3.50 ± 0.62	3.17 ± 0.79	3.5–10.0	0.566	0.772
Lymphocytes (%)	45.83 ± 4.53	52.17 ± 3.03	48.00 ± 4.04	45.0–70.0	0.504	0.919
Biochemical parameters Nunes*et al*. [53]						x
GLU (mmol/L)	11.37 ± 0.46	12.15 ± 0.54	11.63 ± 0.38	10.0–18.0	0.474	0.913
TC (mmol/L)	3.68 ± 0.13	3.52 ± 0.26	3.67 ± 0.12	2.22–5.17	0.821	0.999
TP (g/L)	30.84 ± 1.10^a^	25.49 ± 1.41^b^	27.92 ± 1.29^a^	25.64–47.92	0.0247	0.2687
ALB (g/L)	11.28 ± 1.58	13.55 ± 0.59	13.74 ± 0.55	11.26–21.40	0.291	0.239
ALP (U/L)	3686.09 ± 88.92^a^	3143.20 ± 122.0^a^	2766.13 ± 236.09^b^	711–7432	0.076	0.003
TRIG (mmol/L)	0.08 ± 0.02	0.15 ± 0.03	0.12 ± 0.02	0.18–1.17	0.175	0.524
P (mmol/L)	3.07 ± 0.11^a^	0.54 ± 0.03^b^	0.49 ± 0.06^b^	1.27–3.97	0.0	0.0
Ca (mmol/L)	3.00 ± 0.11	2.98 ± 0.12	3.25 ± 0.07	0.085–2.75	0.987	0.222
GGT (U/L)	23.07 ± 1.68	19.31 ± 1.54	23.38 ± 5.17	1.45–97.51	0.701	0.997

*Data are presented as means ± SD, ^a,b^different letters for the same indicator indicate significant differences between the groups, (p ≤ 0.05). SD=Standard deviation, GLU=Glucose, TC=Total cholesterol, TP=Total protein, ALB=Albumin, ALP=Alkaline phosphatase, TRIG=Triglyceride, P*=*Phosphorus, Ca=Calcium, GGT=Gamma-glutamyltransferase

The NaHum additive contributed to a significant (p < 0.05) decrease in the levels of TP, ALP, and P and a numerical decrease in total TC and GGT levels in blood, compared with the control group. The highest intake rate of NaHum (50 mL/L) caused an increase in the amounts of ALB and Ca (Table-6), which was not statistically significant.

During the research, diarrhea was not clinically observed in the broilers. At the end of the research, the ileal microbiota count (Table-7) showed a slight increase in the *Enterobacteriaceae* count in research group broilers, and the count of *Lactobacilli* was not affected significantly by treatments (8.67 log colony-forming unit [CFU]/g and 8.59 log CFU/g in Group 1 and 2 broilers, respectively), compared with the control group (8.71 log CFU/g).

**Table-7:** Effects of the NaHum feed additive on the ileum microbiota composition of broilers at day 42 (colony-forming unit/g).

Parameters	Control group (n = 12)	Group 1 (n = 12)	Group 2 (n = 12)	p-value
*Enterobacteriaceae*	1.1 × 10^7^± 7.58 × 10^6^	1.2 × 10^7^± 8.16 × 10^6^	1.0 × 10^8^± 1.17 × 10^7^	NA
*Lactobacillus*spp.	5.2 × 10^8^± 1.41 × 10^8^	4.7 × 10^8^± 3.03×10^8^	3.9 × 10^8^± 2.55 × 10^8^	NA
*Clostridium*spp.	7.0 × 10^2^± 0.82	4.3 × 10^2^± 2.26×10^2^	2.5 × 10^2^± 2.30 × 10^2^	NA

*Data are presented as means ± SD. SD=Standard deviation, NA=Not applicable NaHum=Sodium humate

## Discussion

Proper provision of feed and water to poultry is important for their growth, health, and productivity [54–56]. The most common problem with industrially produced feed for poultry is nutrient deficiencies and not adding biologically active principles, or adding them in wrong proportions. Without a balanced diet, poultry have poor plumage, slow development and growth, obesity, and leg problems [57, 58]. Therefore, it is important to seek innovative solutions to find opportunities to use conventional poultry feed more efficiently; besides, it is also important that the feed is made of local raw materials that stimulate economic development in rural areas, thereby providing jobs and incomes for their residents.

One of the most important sources of biologically active principles is freshwater sapropel. Sapropel, depending on the depth, has different agrochemical and physical properties [59]. Therefore, several research studies have tested the possibilities of supplementing complex poultry feed with organic sapropel to increase feed absorption efficiency and develop natural, safe and locally-sourced feed for young poultry [28, 29, 38, 60, 61].

Diets for broiler chickens should aim to achieve a leaner carcass, reducing the feed conversion ratio, and increasing weight gain. Some feed nutrients should also be limited to prevent problems associated with high growth rates at the early life stage of broiler chicks, for example, increased fat deposition, frequent metabolic disorders, increased mortality, and frequent skeletal diseases [62]. In producing Ross 308 broilers, the goal is to reduce the feed conversion ratio from 1.62 to 1.53 for a broiler weighing an average of 2.5 kg. This study showed that the FCR for the research groups was comparable with the desired rate for Ross 308.

In intensive meat poultry farming, farms need to achieve maximum output in the shortest possible period and with the least possible feed consumption. In such farms, broilers are sold for meat at the age of 5–6 weeks, and this is mainly associated with the live weight, breast musculature size, low feed consumption per kg of carcass weight, and fast feathering [63]. In 2013, the worldwide average feed consumption per kg of carcass weight was approximately 2.8 kg [64]. A decrease in live weight gain per day after day 35 could be explained by a decrease in the growth of broiler muscles and more intense development of broiler genitals and bone tissue. Therefore, further fattening of broilers (after day 35) is no longer efficient and economically feasible. This study established that the selling age of broilers (day 36) was fully consistent with that identified by the aforementioned research studies.

Various research studies have found that humic substances have a beneficial effect on the gut microflora of broilers and increase nutrient digestibility, resulting in higher productivity [65, 66]. For example, a research study on the inclusion of humus substances in diets for Ross 308 broiler chickens conducted by El Kaya and Tuncer [67] established a higher weight gain and a lower feed conversion ratio in the research group of broiler chickens (1.68), which was 0.18 higher than that found in this study [67]. A research study conducted in Russia on Ross 308 broiler chickens whose diets were supplemented with humic acid 1 g, 1.5 g, and 2 g found that on day 38, the CY was in the range of 70.5%–73.0% [68]. Kocabagli *et al*. [25] established that adding Farmagulator DRY Humate to broiler feed led to a higher carcass weight and yield (73.47%–74.18%) in the groups fed with the humic additive. This study found that the CY in the research groups was in the range of 79.22–79.24%, which exceeded the CY found in the control group by 2 percentage points. It is also stated that sapropel as a source of humic substances in dry form (3% of the total feed) can increase the average live weight of broilers [28]. Including a sapropel additive in poultry diets increased the gross weight gain of broiler chickens by 1.7%–2.0%, while reducing FC per kg of weight gain by 0.5% [69], which is consistent with the results of this study.

It is important to note that humic substances could also be added to drinking water for poultry, thereby achieving similar positive results. For example, research on laying hens have found that adding humic acids to drinking water increased feed conversion and laying intensity [69]. However, Ozturk *et al*. [70] found that because of an increased rate of intake of the humate additive (1.5% of the total amount of drinking water), the live and carcass weights of broilers decreased and the amount of their intramuscular adipose tissue increased. As a result, a direct negative effect was observed on feed intake capacity and nutritional energy balance, which decreased the yield of important parts of the carcass (breast muscles and thighs) being in demand. However, Ozturk *et al*. [70] found an increase in live weight and more efficient feed absorption in the research group fed with less humic substances (0.5%–1.0% of the total amount of drinking water). Hassan [71] also found that the total live weight and the average weight gain of broiler chickens (Ross 308) were lower in the group fed a humate additive at an intake rate of 10 g/kg of feed than in the group fed with no humates or with a humate additive at a lower intake rate (5 g/kg of feed). The research study by Hassan [71] found that a humate additive (5 g and 10 g/kg of feed) made a significant negative effect on feed conversion in broilers and the broiler performance index, which is calculated as the ratio of live weight to the FCR, expressed in percent relative to the control group.

The most widely used part of the broiler carcass worldwide is the breast muscle, and the demand for it continues to grow due to the high protein and low-fat content. Thigh meat is also in demand, especially if it is deboned and used to prepare various food products. In broiler production, the emphasis is placed on the quality and quantity of the main parts of the carcass (breast fillet, thighs, and legs without bones). Research study conducted in Poland on the inclusion of halloysites in diets for Ross 308 broiler chickens revealed that the proportion of breast musculature was, on average, 31%, while leg musculature was 20% [72]. Some research studies have found that humate additives also increased the weight of broiler carcass parts important for the consumer market. Jadďuttová *et al*. [73] found that adding 8 g and 10 g of humic substances per kg of feed mixture to diets for COBB 500 broiler chickens resulted in significantly higher breast muscle (tenderloin) and thigh weight. A research study conducted in Slovakia on Ross 308 broiler chickens fed with a humate additive revealed that the proportions of heart and liver at the age of 42 days was on average 0.64% and 2.01%, respectively [74], whereas this study found that the proportions in research Groups 1 and 2 were slightly higher but relatively lower than those in the control group. This proves that adding humus additives to the diet improves liver and kidney function. Usually, the absolute count of microbiota in the small intestine is relatively low, on average 10^5^ CFU/g of digesta, and 50% of the total ileal microbiota consists of up to 5 genera; the main colonizing bacteria in the small intestine are usually *Lactobacillus, Enterococcus, Turicibacter*, and *Clostridium* [75]. This study did not find any significant difference in the numbers of ileum microbiota representatives in broilers; however, a 50 mL NaHum additive contributed to a slight increase in the *Enterobacteriaceae* count and a decrease in the *Lactobacillus* count compared with the control group. This could be explained by possible changes in the pH level in the intestine caused by the NaHum additive, and the subsequent changes in the representatives of the microbiota, since the ileal microbiota can sometimes be mixed with the microbiota of cecal origin [76].

This study found a significant decrease in the count of heterophils or granular leukocytes in the blood of the research groups. Heterophils are pronounced phagocytes and can provide a broad spectrum of antibacterial activity. To perform this activity, heterophils use receptor mechanisms to detect and destroy pathogenic bacteria. An increase in the count of heterophils is caused by the activation of the immune system of broilers because of their exposure to microorganisms and mycotoxins. Interleukin (IL)-2 and IL-8 are involved in this process [77]. The results of this study on reduction in the count of heterophils in the blood of research group broilers therefore indicate a reduction in the microbiological risk in the broiler body caused by the NaHum additive.

This study found an increase in the count of eosinophils in the blood of research group broilers, yet it did not necessarily exceed the reference level. Eosinophils mobilize at the site of antigen and antibody response, and this mobilization leads to an increase in the count of eosinophils in circulation [78]. It is likely that the results of this research indicated the activation of the immune system of broilers caused by the NaHum additive. This was also confirmed by the higher count of eosinophils found in research Group 2 than in research Group 1.

Poultry blood proteins serve as an important indicator of health status and allow identification of metabolic changes in the broiler body. These changes are more dynamic in young poultry and are usually associated with intensive metabolic processes and changes in feed intake. The results of a research study conducted in Slovakia [79] showed that significant changes in the proportion of individual protein fractions were found in broilers during intensive development and fattening. These changes were explained by the intensive development of broilers, which was also influenced by different feed components. This study confirmed that the inclusion of the NaHum feed additive in diets for broilers, resulting in a higher live weight gain in the research group, has caused a decrease in the TP level in the blood of broilers (25.49 ± 1.41 g/L in research Group 1 and 27.92 ± 1.29 g/L in research Group 2), while not violating the aforementioned [52] reference level of TP in blood at 25.64–47.92 g/L. However, this study’s data showed an increase in the level of another blood serum protein fraction, ALB, which confirmed the beneficial effect of the NaHum additive on the composition of blood proteins and the functionality of the liver and kidneys. Albumin is the main blood plasma protein responsible for maintaining the osmotic pressure in blood and provides a transport function for various small blood molecules, including fatty acids and bile pigments [80]. After examining the effect of a humic acid feed additive on the health of broilers, this study concluded that a decrease in P levels in the blood was observed when NaHum was added to the drinking water.

It is likely that the decreased blood P levels in broilers are due to the metal chelating effect of humic acid, which is influenced by a large number of carboxylic acid side-chains [81]. Since NaHum was ingested by the broilers with drinking water (rather than in powder form), this might have further contributed to more complete absorption of the alkaline humate additive by the broilers, as the transition time of digesta from ingestion to excretion in the broilers is as short as 2 h [82]. In addition, the research also found a lower proportion of bone tissue in the broiler group watered with NaHum. In another dietary experiment of ours, in which a sapropel (dry form) was added to the feed mixture, the blood P level in the broilers was higher (in the range of 3.95–4.25 mmol/L), slightly exceeding that found in the control group (on average 3.07 mmol/L) (unpublished data).

## Conclusion

The research results show that NaHum extracted from freshwater sapropel is a promising and versatile material as a dietary supplement in poultry farming; the extraction is a relatively simple and environmentally friendly process that can be easily scaled up for industrial production. The dietary research with Ross 308 one-day-old broiler chicks found a positive effect of NaHum on the performance of broiler chickens; as feed intake per unit of production decreased, the live weight of broiler chickens increased, higher productivity was achieved, and the economic efficiency and cost of broiler production increased, i.e. feed conversion rate was the same in both research groups in terms of both live weight gain and carcass weight. However, the average carcass weight was significantly higher in both research groups. The NaHum additive modulates the ileum microbiota and metabolic processes in the broiler body. Considering the positive effects of NaHum derived from freshwater sapropel on the productivity and economic efficiency of broiler chickens, the NaHum feed additive should be further investigated on commercial farms on a larger scale to obtain the results that could be reasonably used in practice, as this study was based on a small number of research poultry. Because this study has not found any references in existing scientific literature to decreased blood P levels in broilers or other farm animals after adding liquid NaHum to their drinking water, we believe that it is important to continue the research to draw reasonable conclusions on the effects of NaHum in liquid form on the health performance of farm animals.

## Authors’ Contributions

LP: Conceptualization and validation, methodology, investigation, visualization, data analysis, formal analysis, writing of the original manuscript. DB: Methodology, sample collection, investigation, visualization, data analysis. IP: Conceptualization and validation, project administration and funding acquisition, supervision, writing-review and editing. SC: Visualization, software and resources, methodology, writing-review and editing. AV: Methodology, investigation, sample collection. IV: Methodology, investigation, sample collection, data analysis, visualization, resources. SM: Methodology, investigation. All authors have read and agreed to the published version of the manuscript.

## References

[1] Hobbs J.E (2021). The Covid-19 pandemic and meat supply chains. Meat Sci.

[2] Andretta I, Hickmann F.M.W, Remus A, Franceschi C.H, Mariani A.B, Orso C, Kipper M, Létourneau-Montminy M.P, Pomar C (2021). Environmental impacts of pig and poultry production:Insights from a systematic review. Front. Vet. Sci.

[3] Boland M.J, Rae A.N, Vereijken J.M, Meuwissen M.P.M, Fischer A.R.H, van Boekel M.A.J.S, Rutherfurd S.M, Gruppen H, Moughan P.J, Hendriks W.H (2013). The future supply of animal-derived protein for human consumption. Trends Food Sci. Technol.

[4] Pretty J (2008). Agricultural sustainability:Concepts, principles and evidence. Philos. Trans. R. Soc. Lond. B Biol. Sci.

[5] Moekti G.R (2020). Industrial livestock production:A review on advantages and disadvantages. IOP Conf. Ser. Earth Environ. Sci.

[6] Burton E, Scholey D, Alkhtib A, Williams P (2021). Use of an ethanol bio-refinery product as a soy bean alternative in diets for fast-growing meat production species:A circular economy approach. Sustainability.

[7] Restoux G, Rognon X, Vieaud A, Guemene D, Petitjean F, Rouger R, Brard-Fudulea S, Lubac-Paye S, Chiron G, Tixier-Boichard M (2022). Managing genetic diversity in breeding programs of small populations:The case of French local chicken breeds. Genet. Sel. Evol.

[8] Costa M.M, Alfaia C.M, Lopes P.A, Pestana J.M, Prates J.A.M (2022). Grape by-products as feedstuff for pig and poultry production. Animals (Basel).

[9] Falcon W.P, Naylor R.L, Shankar N.D (2022). Rethinking global food demand for 2050. Popul. Dev. Rev.

[10] Castro F.L.S, Chai L, Arango J, Owens C.M, Smith P.A, Reichelt S, DuBois C, Menconi A (2023). Poultry industry paradigms:Connecting the dots. J. Appl. Poult. Res.

[11] Dohlman E, Hansen J, Boussios D (2022). USDA Agricultural Projections to 2031, USDA Miscellaneous 323859.

[12] Gozali R.M (2020). Industrial livestock production:A review on advantages and disadvantages. IOP Conf. Ser. Earth Environ. Sci.

[13] Nikos A, Bruinsma J (2012). World Agriculture towards 2030/2050:The 2012 Revision. ESA Working Paper No. 12-03.

[14] European Commission (2022). Short-term Outlook for EU Agricultural Markets.

[15] Lukić M, Petričević V, Delić N, Tolimir N, Dosković V, Rakonjac S, Škrbić Z (2022). How does the choice of genotype and feed in the local market affect broiler performance and the farm economy?A case study in Serbia. Agriculture.

[16] Khan N.A, Ali M, Ahmad N, Abid M.A, Kusch-Brandt S (2022). Technical efficiency analysis of layer and broiler poultry farmers in Pakistan. Agriculture.

[17] Bareith T, Csonka A (2022). Dynamics of competition in the Hungarian poultry industry. AGRIS Online Pap. Econ. Inform.

[18] Association of Poultry Processors and Poultry Trade in the EU Countries (AVEC) (2022). Annual Report 2022.

[19] Parolini M, Ganzaroli A, Bacenetti J (2020). Earthworm as an alternative protein source in poultry and fish farming:Current applications and future perspectives. Sci. Total Environ.

[20] Orheruata A.M, Nwokoro S.O, Alufohai G.O, Omagbon B.I (2006). Growth indices and economy of feed intake of broiler chickens fed changing commercial feed brands at starter and finisher phases. Int. J. Poult. Sci.

[21] Siegert W, Zuber T, Sommerfeld V, Krieg J, Feuerstein D, Kurrle U, Rodehutscord M (2019). Prececal amino acid digestibility and phytate degradation in broiler chickens when using different oilseed meals, phytase and protease supplements in the feed. Poult. Sci.

[22] Proskina L, Cerina S, Valdovska A, Pilvere I, Alekneviciene V (2021). The possibility of improving meat quality by using peas and faba beans in feed for broiler chickens. Potr. S. J. F. Sci.

[23] Arif M, Alagawany M, Abd El-Hack M.E, Saeed M, Arain M.A, Elnesr S.S (2019). Humic acid as a feed additive in poultry diets:A review. Iran. J. Vet. Res.

[24] Eren M, Gezen Ş.Ş, Deniz G, Türkmen I.I (2000). Effects of humates supplemented to the broiler feeds on fattening performance, serum mineral concentration and bone ash/Broyler yemlerine katılan duma tlarin besi performansı, serum mineral konsantrasyonu ve kemik külüüzerine etkileri. Ankara Üniv. Vet. Fakült. Derg.

[25] Kocabagli N, Alp M, Acar N, Kahraman R (2002). The Effects of Dietary Humate Supplementation on Broiler Growth and Carcass Yield. Poult. Sci.

[26] Karaoglu M, Macit M, Esenbuga N, Durdag H, Turgut L, Bilgin O.C (2004). Effect of supplemental humate at different levels on the growth performance, slaughter and carcass traits of broilers. Int. J. Poult. Sci.

[27] Murunga S.I, Wafula E.N, Sang J (2020). The use of freshwater sapropel in agricultural production:A new frontier in Kenya. Adv. Agric.

[28] Kuzmina V.V, Skvortsova E.G, Pivovarova E.A, Bushkareva A.S, Vostrova U.A, Poltoratskaya A (2021). Influence of sapropel on the activity of intestinal peptidases of broiler chickens. J. Indones. Trop. Anim. Agric.

[29] Vanadziņš I, Mārtiņsone I, Kļaviņa A, Komarovska L, Auce A.C, Dobkeviča L, Sprūdža D (2022). Sapropel-mining characteristics and potential use in medicine. Proc. Latvian Acad. Sci. Sec. B Nat. Exact Appl. Sci.

[30] Dmitriyeva E.D (2003). Chemical Composition and Biological Activity of Sapropel from Belgorod Region. Doctoral Thesis.

[31] The European Landowners'Organization (ELO) (2022). Joint Statement 27^th^ January 2022 Farm to Fork Strategy:How to Reach the Targets?.

[32] Bezuglova O, Klimenko A (2022). Application of humic substances in agricultural industry. Agronomy.

[33] De Lourdes Angeles M, Gómez-Rosales S, Téllez-Isaias G (2022). Mechanisms of action of humic substances as growth promoters in animals. Humus and Humic Substances-Recent Advances.

[34] Kamel M.M, Elhady M.M, El Iraqi K.G, Wahba F.A (2015). Biologyical immune stimulants effects on immune response, behavioural and productive performance. Egypt. Poult. Sci. J.

[35] Islam K.M.S, Schumacher A, Gropp J.M (2005). Humic acid substances in animal agriculture. Pak. J. Nutr.

[36] Trckova M, Matlova L, Hudcova H, Faldyna M, Zraly Z, Dvorska L, Beran V, Pavlik I (2005). Peat as a feed supplement for animals:A review. Vet. Med.

[37] Hafsa S.H, Hassan A.A, Sabek A, Elghandour M.M.M.Y, Barbabosa-Pliego A, Alqaisi O, Salem A.Z.M (2021). Extracted and characterized humic substances as feed supplement in rabbit feeding:Effects on performance, blood metabolites and caecal fermentation activity. Waste Biomass Valor.

[38] Morozov V.V, Bogdanov K.A, Ignatenkov V.G (2021). The influence of design parameters on the pressure in the extruder for the production of sapropelic feed. IOP Conf. Ser. Earth Environ. Sci.

[39] Butka M, Latvietis J, Kamphues J, Falachowsky G (2001). Lake Sapropel Additive into Layer Feed. Conference Workshop 6 on Sustainable Animal Production, Hannover, Germany, 2000. Animal Nutrition-Resources and New Tasks.

[40] LR likums “Par Eiropas Konvenciju par Lauksaimniecības Dzīvnieku Aizsardzību un Tās Protokolu”. Latvijas Vēstnesis 72, 05.05.2007. Latvijas Republikas Saeimas un Ministru Kabineta Ziņotājs 11, 14.06.2007 (Law of the Republic of Latvia “On the European Convention for the Protection of Animals Kept for Farming Purposes and the Protocol”. Latvian Herald 72, 05/05/2007 Herald of the Parliament and the Cabinet of the Republic of Latvia, 11, 14/06/2007) (in Latvian).

[41] Directive 2010/63/EU of the European Parliament and of the Council of 22 September 2010 on the Protection of Animals Used for Scientific Purposes (Text with EEA Relevance) Official Journal of the European Union, OV, L.

[42] Kurzo B, Hajdukiewicz O, Krasnoberskaya O (2004). Relationships of sapropel formation in lake-mire complexes of Belarus. Limnol. Rev.

[43] Latvian Environment Geology, Meteorology Centre/Latvijas Vides Ģeoloġijas un Metroloġijas Centrs (2022). Summary Report on Mineral Resources (Construction Materials Raw Materials, Peat, Sapropel and Healing Sludge) Gains, Stocks and their Changes [Kopsavilkuma Pārskats par Derīgo Izrakteņu (Būvmateriālu Izejvielu, Kūdras, Sapropeļa un Dziedniecības Dūņu) Ieguvi, Krājumiem un to Izmaiņām. p16. (in Latvian)].

[44] Segliņš V (2013). The most important results and perspectives of the “Deeps of the Earth”project of the state research program [Valsts pētījumu programmas projekta “Zemes dzīles”svarīgākie rezultāti un perspektīvas (in Latvian)]. Mater. Sci. Appl. Chem.

[45] Stankeviča K, Kļaviņš M (2013). Sapropel and possibilities of its use [Sapropelis un tāizmantošanas iespējas (in Latvian)]. Mater. Sci. Appl. Chem.

[46] State Office of Environmental Supervision (2022). Opinion No. 5-04/2/2022 Regarding the Environmental Impact Assessment Report of the Mining of Sapropel in Biža Lake, Andrupene Parish, Krāslava County.

[47] Sarlaki E, Paghaleh A.S, Kianmehr M.H, Vakilian K.A (2020). Chemical, spectral and morphological characterization of humic acids extracted and membrane purified from lignite. Chem. Chem. Technol.

[48] Official Journal of the European Union (2007). Directive 2007/43/EC of the European Parliament and of the Council of 28 June 2007 Laying Down Minimum Rules for the Protection of Chickens Kept for Meat Production (Text with EEA Relevance), Current Consolidated Version:14/12/2019. Official Journal of the European Union 182, p19–28.

[49] Aviagen (2018). Information library. Ross Broiler Management Handbook.

[50] Marcu A, Vacaru-Opriş I, Dumitrescu G, Petculescu L.C, Marcu A, Nicula M, Peţ I, Dronca D, Kelciov B, Mariş C (2013). The influence of genetics on economic efficiency of broiler chickens'growth. Sci. Pap. Anim. Sci. Biotechnol.

[51] Martins J.M.S, Carvalho C.M.C, Litz F.H, Silveira M.M, Moraes C.A, Silva M.C.A, Fagundes N.S, Fernandes E.A (2016). Productive and economic performance of broiler chickens subjected to different nutritional plans. Bras. Cienc. Avic.

[52] Jain N.C (1993). Essential of Veterinary Hematology.

[53] Nunes V, Broch J, Wachholz L, de Souza C, Damasceno J.L, Oxford J.H, Bloxham D.J, Billard L, Pesti G.M (2018). Choosing sample sizes for various blood parameters of broiler chickens with normal and non-normal observations. Poult. Sci.

[54] Davis M.J (2021). Top Tips for Feed and Water Management in Broiler Houses. Poultry Health and Disease/Broiler Husbandry/Poultry Nutrition. WATT Poultry.

[55] Fairchild B.D, Ritz C.W (2015). Poultry Drinking Water Primer. UGA Extension Bulletin 1301.

[56] Esmail S.H (2022). Factors Affecting Water Intake and Its Utilisation by Chickens. Poultry World.

[57] Arrazola A, Torrey S (2021). Welfare and performance of slower growing broiler breeders during rearing. Poult. Sci.

[58] Skinner-Noble D.O, Teeter R.G (2003). Components of feed efficiency in broiler breeding stock:energetics, performance, carcass composition, metabolism, and body temperature. Poult. Sci.

[59] Morozov V, Savelyeva L, Nesterova E (2020). Justification of production indicators of organic fertilizer based on sapropel. IOP Conf. Ser. Mater. Sci. Eng.

[60] Mikulioniene S, Baležentiene L (2012). Effectiveness and potential usefulness of dietary supplementation with sapropel on ducklings and goslings growth and quality indices. Vet. Ir Zootech.

[61] Yurina N, Khorin B, Yurin D, Semenenko M, Kuzminova E (2020). The effect of feeding a natural feed additive on the performance of broiler chickens. E3S Web Conf.

[62] Smith E.R, Pesti G.M, Bakalli R.I, Ware G.O, Menten J.F (1998). Further studies on the influence of genotype and dietary protein on the performance of broilers. Poult. Sci.

[63] Nematbakhsh S, Selamat J, Idris L.H, Razis A.F.A (2021). Chicken authentication and discrimination via live weight, body size, carcass traits, and breast muscle fat content clustering as affected by breed and sex varieties in Malaysia. Foods.

[64] Alexander P, Brown C, Arneth A, Finnigan J, Rounsevell M.D (2016). Human appropriation of land for food:The role of diet. Glob. Environ. Change.

[65] Taklimi S.M.S.M, Ghahri H, Isakan M.A (2012). Influence of different levels of humic acid and esterified glucomannan on growth performance and intestinal morphology of broiler chickens. Agric. Sci.

[66] Arif M, Rehman A, Saeed M, Abd El-Hack M.E, Arain M.A, Haseebarshad M, Zakria H.M, Abbasi I.H (2016). Impacts of dietary humic acid supplementation on growth performance, some blood metabolites and carcass traits of broiler chicks. Indian J. Anim. Sci.

[67] El Kaya C.A, Tuncer S.D (2009). The effects of humates on fattening performance, carcass quality and some blood parameters of broilers. J. Anim. Vet. Adv.

[68] Simakova I.V, Vasiliev A.A, Korsakov K.V, Sivokhina L.A, Salautin V.V, Gulyaeva L.Y, Dmitriev N.O (2021). Role of humic substances in formation of safety and quality of poultry meat. Humic Substances.

[69] Arafat R.Y, Khan S.H, Abbas G, Iqbal J (2015). Effect of dietary humic acid via drinking water on the performance and egg quality of commercial layers. Am. J. Life Sci.

[70] Ozturk E, Ocak N, Coskun I, Turhan S, Erener G (2009). Effects of humic substances supplementation provided through drinking water on performance, carcass traits and meat quality of broilers. J. Anim. Physiol. Anim. Nutr.

[71] Hassan S.M (2014). Effect of adding dietary humate on productive performance of broiler chicks. Asian J. Poult. Sci.

[72] Banaszak M, Biesek J, Adamski M (2022). Research note:Growth and meat features of broiler chicken with the use of halloysite as a technological additive to feed and peat litter. Poult. Sci.

[73] Jadďuttová I, Marcinčáková D, Bartkovský M, Semjon B, Harčárová M, Nagyová A, Váczi P, Marcinčák S (2019). The effect of dietary humic substances on the fattening performance, carcass yield, blood biochemistry parameters and bone mineral profile of broiler chickens. Acta Vet. Brno.

[74] Hrnčár C, Nikolova N, Bujko J (2018). The effect of single and combined use of probiotic and humate on fattening performance, carcass characteristics and internal organs of broiler chickens. Maced. Vet. Rev.

[75] Kollarcikova M, Kubasova T, Karasova D, Crhanova M, Cejkova D, Sisak F, Rychlik I (2019). Use of 16S rRNA gene sequencing for prediction of new opportunistic pathogens in chicken ileal and cecal microbiota. Poult. Sci.

[76] Yan W, Sun C, Zheng J, Wen C, Ji C, Zhang D, Chen Y, Hou Z, Yang N (2019). Efficacy of fecal sampling as a gut proxy in the study of chicken gut microbiota. Front. Microbiol.

[77] Kogut M.H, Genovese K.J, Lowry V.K (2001). Differential activation of signal transduction pathways mediating phagocytosis, oxidative burst, and degranulation by chicken heterophils in response to stimulation with opsonized *Salmonella* enteritidis. Inflammation.

[78] Agboola A.F, Omidiwura B.R, Olurinola J.O (2017). Influence of four dietary oils on selected blood constituents in egg-type chickens. J. Agric. Sci. Belgrade.

[79] Tóthová C, Sesztáková E, Bielik B, Nagy O (2019). Changes of total protein and protein fractions in broiler chickens during the fattening period. Vet. World.

[80] Adriani L, Mushawwir A, Kumalasari C, Nurlaeni L, Lesmana R, Rosani U (2021). Improving blood protein and albumin level using dried probiotic yogurt in broiler chicken. Jordan J. Biol. Sci.

[81] Rath N.C, Huff W.E, Huff G.R (2006). Effects of humic acid on broiler chickens. Poult. Sci.

[82] Hughes R.J (2008). Relationship between digesta transit time and apparent metabolisable energy value of wheat in chickens. Br. Poult. Sci.

